# Investigating the relationship between melatonin levels, melatonin system, microbiota composition and bipolar disorder psychopathology across the different phases of the disease

**DOI:** 10.1186/s40345-019-0163-y

**Published:** 2019-12-09

**Authors:** Mirko Manchia, Alessio Squassina, Claudia Pisanu, Donatella Congiu, Mario Garzilli, Beatrice Guiso, Federico Suprani, Pasquale Paribello, Vittoria Pulcinelli, Maria Novella Iaselli, Federica Pinna, Flavia Valtorta, Bernardo Carpiniello, Stefano Comai

**Affiliations:** 1 Section of Psychiatry, Department of Medical Sciences and Public Health, University of Cagliari, Cagliari, Italy; 2 Unit of Clinical Psychiatry, University Hospital Agency of Cagliari, Cagliari, Italy; 3 Department of Pharmacology, Dalhousie University, Halifax, NS Canada; 4 Department of Biomedical Science, Section of Neuroscience and Clinical Pharmacology, University of Cagliari, Cagliari, Italy; 5 San Raffaele Scientific Institute and Vita Salute University, Via Olgettina 58, 20132 Milan, Italy; 6 Department of Psychiatry, McGill University, Montreal, QC Canada

**Keywords:** Melatonin, Bipolar disorder, Genetic variability, Microbiota, Observational study

## Abstract

**Background:**

Bipolar disorder (BD) is characterized by recurrent episodes of depression and mania/hypomania alternating with intervals of well-being. The neurobiological underpinnings of BD are still veiled although there is evidence pointing to a malfunction of the circadian clock system that is regulated by the neuromodulator melatonin (MLT). Small sample size studies in BD patients have shown that changes in the levels of MLT are associated with shifts in illness status. Moreover, mood stabilizers (including lithium and valproic acid) influence the MLT system. Of interest, MLT also modulates intestinal microbiota, and recent work suggests an important role of microbiota alterations in neuropsychiatric disorders, including BD. This study is designed to explore whether the possible patterns of associations between changes in the levels of MLT and its precursors and BD mood phases are modulated by variants within the genes encoding for the elements of the MLT system and/or by the microbiota composition.

**Methods:**

We will conduct a 2-year follow-up study in 50 BD patients during the three different mood phases of the disease. For each phase, we will perform a blood withdrawal for the analysis of MLT levels and of variants of the genes related to the MLT pathway between 8 and 10 a.m. after an overnight fasting, a stool specimen collection for the analysis of microbiota composition, and a detailed psychometric assessment for depression, mania, impulsivity and cognitive abilities. We will also recruit 50 healthy age-matched controls in whom we will perform a blood withdrawal between 8 and 10 a.m. after an overnight fasting, a stool specimen collection, and a psychometric assessment to exclude the presence of psychiatric disorders.

**Discussion:**

In this cross sectional (case–control vs. BD comparisons) and longitudinal (24 months) study, we expect to clarify the link between the MLT system, microbiota and BD psychopathology. We expect to identify some typical BD symptomatic clusters that will be more strictly associated with variations in the MLT system. In a personalized medicine perspective, this subgroup of BD patients may benefit from a pharmacological therapy targeting the MLT system.

*Trial registration* This study protocol was approved by the Ethics Committee of the University Hospital Agency of Cagliari (PG/2019/6277)

## Background

Bipolar disorder (BD) is a complex chronic psychiatric condition characterized by recurrent episodes of depression and mania/hypomania alternating with intervals of well-being. According to the World Health Organization, BD is among the top 10 leading cause of years lived with disability (Murray and Lopez 1996). Epidemiological estimates show that 1–5% of people across all populations suffers from diverse types of BD (Angst et al. 2003). Further, BD carries a high burden in terms of treatment costs (Dilsaver 2011), effect on families and careers, loss of productivity and increased mortality (Lomholt et al. 2019). Patients with BD experience substantial residual morbidity resulting in up to 50% of follow-up time spent ill, mainly in depression (Joffe et al. 2004; Post et al. 2003), with a risk of relapse at 5 years that remains high (about 70%) even in the presence of maintenance treatment (Gitlin et al. 1995). Selecting the most effective therapy for a BD patient thus still remains a challenge for clinicians (Geddes and Miklowitz 2013) and despite progress in the study of the neurobiology and psychopharmacology of the disease, options remain limited and results are often unsatisfactory. Thus, more research is needed to better understand the neurobiology of the disease and to discover and validate novel potential targets for drug development.

A key component of BD psychopathology (i.e. illness cyclicity) appears to correspond to a neurobiological malfunction of the circadian clock system (Dallaspezia and Benedetti 2009, 2015) that is regulated by the suprachiasmatic nucleus (SCN). Patients affected by BD show alterations of intrinsic biological rhythms, including the sleep–wake cycle, as well as of hormonal rhythms and of the regulation of body temperature (Harvey 2008). A key neurochemical modulator of the circadian clock system is melatonin (MLT). This neuromodulator derives from serotonin (5-HT) and is mainly synthesized in the pineal gland during the night. The physiological effects of MLT are mostly mediated by two high-affinity G-protein coupled receptors (GPCRs), MT_1_ and MT_2_ (Dubocovich et al. 2010; Jockers et al. 2016). MLT is one of the endogenous entraining signals for the circadian system, regulating the activity of the SCN that expresses both MT_1_ and MT_2_ receptors (Dubocovich et al. 2010; Jockers et al. 2016). However, the exact mechanism/s through which MLT regulates the SNC is still to be clarified. Indeed, in vitro and in vivo studies have reported contrasting findings (Dubocovich and Markowska 2005) [for details on this topic, please see our recent papers (Gobbi and Comai 2019a, b)]. The MLT system seems to be a potential target system for the treatment of mood disorders since agomelatine, a non-selective agonist for both MT_1_ and MT_2_ receptors and an antagonist for 5-HT_2C_ receptors has entered clinical use for the treatment of major depression (de Bodinat et al. 2010).

Given the link between MLT and the circadian rhythms, and between the latter and BD (McCarthy 2019; Gonzalez et al. 2018), a series of seminal studies have investigated and demonstrated changes in the levels of MLT associated with illness status in patients with BD (Kennedy et al. 1996; Lam et al. 1990; Nurnberger et al. 2000). In particular, affected subjects have low serum levels of MLT than healthy controls (trait marker) (Kennedy et al. 1996). However, Whalley et al. (1991) did not replicate this finding. Although there is no apparent variation in the MLT levels in relation to a specific phase of the disease (Kennedy et al. 1996), the peak of MLT at night is delayed in euthymic BD patients (Nurnberger et al. 2000). In addition, Nurnberger et al. (2000) showed higher light-induced suppression of MLT in BD patients than in controls, whereas, in contrast, Lam et al. (1990) reported greater light-induced suppression of MLT in controls than in BD patients. Whalley et al. (1991) instead did not find any significant difference in the effect of light on MLT comparing healthy individuals and BD patients (Whalley et al. 1991). Therefore, the presence of a MLT supersensitivity to light as a reliable trait marker of BD needs to be clarified. Overall, the small size of the population analyzed strongly limits the validity of the currently available studies.

In addition to a possible link between dysfunctional MLT levels and BD pathophysiology, there is also a strong hint for a modulating effect of treatments for BD on MLT levels and function. Indeed, in healthy volunteers the mood stabilizer valproic acid significantly decreased the sensitivity of MLT to light (Hallam et al. 2005). Further, in a study in Long Evans rats, lithium significantly influenced MLT levels in several brain regions along the retinal-hypothalamic-pineal pathway (Seggie et al. 1987).

Another biological component, non-genetic but heritable to some extent, that could impact brain function is the gut microbiota. There is growing evidence that the bidirectional signalling along the gut-brain axis may play an important role in the pathophysiology of depression, anxiety and autism spectrum disorder (ASD) (Foster and McVey Neufeld 2013; Sharon et al. 2019; Zheng et al. 2016). Relevant to our project is the evidence that microbiota alterations could modulate the risk of BD, although findings are still preliminary (Nguyen et al. 2018; Pisanu and Squassina 2018). For instance, a diminished representation of Faecalibacterium and of an as yet unclassified member of the Ruminococcaceae family has been detected in subjects with BD with respect to controls (Evans et al. 2017). Interestingly, BD patients with higher levels of Faecalibacterium displayed better sleep quality and less severe depressive symptoms (Evans et al. 2017).

The gut is the main organ involved in peripheral synthesis of MLT, and MLT and its precursor tryptophan exert an important regulatory role on the gastro-intestinal (GI) functions (Bubenik 2002). Indeed, altered tryptophan metabolism in the gut can lead to increased GI permeability, which in turn has been associated with psychiatric disorders (Kelly et al. 2015). MLT, when administered in mice subjected to a high-fat diet (HFD), reduces body weight, hepatic steatosis, low-grade systemic inflammation, improves insulin resistance, and significantly modifies the composition of the intestinal microbiota (Xu et al. 2017). A recent study indicates that MLT improves lipid metabolism in mice with a HFD diet probably through reprogramming of the microbiota (Yin et al. 2018). These still limited and recent findings highlight the importance of the intestinal microbiota in mediating the various physiological functions of MLT.

Collectively, this evidence suggests a potential involvement of MLT and of the MLT system in the physiopathology of BD. The present study protocol is designed to explore in a sample of BD patients the possible link between the MLT system, microbiota and psychopathology across the different mood phases of the illness. In particular, we aim at exploring whether the patterns of associations identified between the levels of MLT and its precursors [tryptophan (Trp), 5-hydroxytryptophan (5-HTP), and 5-HT)] and BD mood phases are modulated by variants within the genes encoding for the elements of the MLT system, including MLT receptors and the enzymes involved in the synthesis and metabolism of MLT, and the microbiota composition.

## Methods/design

### Recruiting procedures

We will perform a 2-year follow-up study in 50 patients affected by BD and in 50 healthy controls of the same age group. Based on the existing data on the natural history of BD and our clinical experience, we estimate that 2 years should be sufficient to evaluate recurrences in BD patients who tend to have cycles of illness that last more than 1 year. A prospective follow-up with an in person psychiatric assessment and administration of psychometric scales will be done every month up to 24 months. In addition, weekly telephone assessments will be performed to detect eventual mood fluctuations. During the three different mood phases of the disease, BD patients will undergo, between 8 and 10 a.m. after an overnight fasting, a blood withdrawal, a stool specimen collection, and a detailed psychometric assessment for depression, mania, impulsivity and cognitive abilities. Further, recruited patients will undergo a medical examination to exclude the presence of severe unregulated medical conditions, such as for instance cardiovascular or metabolic diseases. Similarly, between 8 and 10 a.m. after an overnight fasting, control subjects will undergo a clinical assessment to exclude the presence of psychiatric disorders, a medical examination to exclude eventual severe unregulated medical conditions such as cardiovascular or metabolic diseases, a blood withdrawal and a stool specimen collection.

### Diagnostic criteria

The clinical diagnosis of BD will be formulated using the Italian version of the Structured Clinical Interview for DSM 5 (SCID-5) (First et al. 2017).

#### Inclusion criteria for BD patients

- Diagnosis of BD type 1 or type 2 according to the DSM-5 criteria;
- Age between 18 and 65 years old.

#### Exclusion criteria

- Women in the fertile age, who do not use adequate contraception or who are pregnant;
- Past history of traumatic brain insults;
- Diagnosis of current and/or lifetime other psychiatric or neurological disorders or other severe unregulated medical conditions;
- Diagnosis of current and/or lifetime substance use disorder;
- Treatment with melatonergic compounds (melatonin and/or agomelatine) for at least 2 months before enrollment. Other treatments (beta blockers, benzodiazepines, low dose antipsychotics) will be allowed.

#### Inclusion criteria for controls

- Absence of psychiatric disorders diagnosed according to the DSM-5;
- Age between 18 and 65 years old;
- Absence of psychiatric, neurological or other severe unregulated medical conditions.

### Psychometric evaluations

We will perform at each time point an extensive psychometric evaluation using the following validated scales: the Italian version of the Hamilton Depression Rating Scale 21-items (Hamilton 1960), the Italian version of the Hamilton Anxiety Rating Scale 21-item (Hamilton 1959), the Italian version of the Young Mania Rating Scale (YMRS) (Young et al. 1978); the Italian version of the Brief Assessment of Cognition in Affective Disorder (Keefe et al. 2014), the Italian version of the Clinical Global Index of Severity and a measure of the quality of life (WHOQOL-BREF) (Guy 1976), and the Italian version of the Barratt Impulsiveness Scale version-11 (Barratt 1985).

### Sampling procedures

Blood samples will be collected in BD patients in the three different mood phases of the disease (euthymia, depression and hypomania/mania) with a withdrawal performed from 8:00 a.m. to 10:00 a.m. after an overnight fasting. Three 10 mL aliquots (one for the analysis of the plasma levels of MLT and its precursors and two for genetic analyses) will be collected in EDTA containing tubes and immediately centrifuged at 2500 rpm at 4 °C for 10 min.

### Analysis of the plasma levels of MLT and its precursors

The analysis of plasma levels of MLT and its metabolites will be conducted according to our previously validated method (Longatti et al. 2007; Messaoud et al. 2018) by using an HPLC system (Varian Inc) provided with a Shimadzu RF-10AXL fluorimetric detector. The chromatographic separation is performed at room temperature using a Synergi Fusion-RP 80A column (4 μm; 250 mm × 4.6 mm; Phenomenex, Aschaffenburg, Germany). The mobile phase consists of acetonitrile/phosphate buffer (0.004 M, pH 3.5) in isocratic elution (15:85 v/v) and with a flow rate of 0.9 ml/min. The fluorimetric detector is placed at the excitation and emission wavelengths of 285 and 345 nm, respectively. Plasma samples are diluted 1:10 before the chromatographic analysis. The determinations will be conducted in triplicate for each sample analyzed.

### Genetic analysis

Genetic analysis will be performed with the Infinium PsychArray-24 BeadChip 1.2 (Illumina, San Diego, California, USA). This chip allows the evaluation of the genetic variants associated with the most common psychiatric disorders and includes 271,000 polymorphisms of the Infinium Core-24 BeadChip, 277,000 markers of the Infinium Exome-24 BeadChip, and 50,000 additional markers associated with common psychiatric disorders. Furthermore, the Infinium PsychArray-24 + v1.2 enables to add up to 60,000 additional markers, which in our study will be dedicated to the genes involved in the MLT pathway. The following genes related to the MLT pathway will be studied: MT_1_ receptor (*MTNR1A*); MT_2_ receptor (*MTNR1B*); tryptophan hydroxylase 1; tryptophan hydroxylase 2; acetylserotonin *O*-methyltransferase; aralkylamine *N*-acetyltransferase; retinoid-related orphan nuclear receptor alpha; retinoid-related orphan nuclear receptor beta; calmodulin 1; calmodulin 2; calmodulin 3; serotonin transporter (SLC6A4); catechol *O*-methyltransferase; monoamine oxidase A. Quality control and association analyses for the PsychArray will be carried out using PLINK v. 1.9 (Chang et al. 2015) applying stringent filtering criteria based on call rate, minor allele frequency, Hardy–Weinberg Disequilibrium, subject call rate. Considering the complexity of the study design and the large number of tests, only variants showing significance after correction for multiple testing in the comparison of patients versus controls will be carried forward in the next steps of the study. These significant variants will be included in regression models to test their effect on the clinical and molecular variables.

### Microbiota analysis

Microbial DNA will be isolated from the fecal material and then the 16S rRNA gene will be amplified. The analysis is completed by sequencing the regions V3 and V4 of the 16S rRNA gene pool corresponding to the microorganisms contained in the microbiota and then by their recognition with the use of bioinformatics tools (Metagenomics Illumina Inc. and Kraken APP). Analysis of the data generated on the Miseq System will be carried out using the BaseSpace 16 S Metagenomics App (Illumina), whereas operational taxonomic unit (OTU) mapping to the Greengenes database will be performed using the Quantitative Insights Into Microbial Ecology (QIIME) platform. Sequences containing ambiguous or low-quality bases will be filtered out using QIIME filter. Remaining sequences will be assigned to each sample according to the unique barcodes. Alpha diversity will be estimated using Shannon index metric.

### Statistical analysis

Differences in plasma levels of MLT and its precursors among the different disease stages will be tested by using repeated measures ANOVA, followed by the Tukey post hoc correction. The Kruskal–Wallis or Friedman test will be used in case data are not normally distributed. We will also compare the levels of MLT and its precursors between BD patients and controls by using t-test or Mann–Whitney U test, depending on the empirical data distribution. We will use regression analyses in order to evaluate the association between levels of MLT and its precursors and the measures of psychopathological severity. Regarding the microbiota analysis for quantitative variables, the indexes of position, dispersion and shape will be measured, while for the categorical ones, frequency of each individual class will be calculated. Data will be analyzed by using packages implemented in the Bioconductor suite in R. Regarding genetic analysis, we will use the chi-square, logistic regression and linear regression tests in order to evaluate the genetic associations, focusing especially on genes encoding elements of the MLT system. All these analyses will be performed using PLINK v. 1.9 (Chang et al. 2015) and R v. 3.6.1 (Team 2014). Each one of the tests performed in every analytical category will be corrected for multiple comparisons depending on the design of the test. However, considering the exploratory nature of this study, statistical significance after correction will not constitute a limitation for the identification and selection of molecular signatures for future investigations.

### Analysis of the statistical power and sample size

Previous estimates (Kennedy et al. 1996) have quantified the magnitude of the effect size of the statistically significant difference in MLT levels between healthy controls and BD patients that was 5.29. Considering this effect size, the analysis of statistical power performed with G*Power program (version 3.1.) established that a sample of 50 BD patients and 50 controls has more than 95% of the statistical power to identify a difference between groups, with an α set at 0.05. According to previous estimates of effect sizes for microbiota analyses (Painold et al. 2019), this sample size should be sufficient to achieve more than 90% of statistical power to detect a difference between groups, even accounting for an attrition rate of 10% during the follow-up with the same α.

### Ethical issues and permission

The study will be conducted in accordance with the recommendations adopted by the 18th World Medical Assembly, Helsinki 1964 and subsequent revisions. Informed consent will be obtained from the patient prior to the enrollment in the study. This study protocol was approved by the Ethics Committee of the University Hospital Agency of Cagliari (PG/2019/6277) on May 8, 2019. All data collected during the study procedures will be processed in accordance with the current regulations for the protection of privacy (regulation (EU) 2016/679 of the European parliament and of the council of 27 April 2016 (General Data Protection Regulation). In order to ensure the confidentiality of the data collected and blood samples, each patient will be given an identification code (a progressive number starting from 1 to 200 followed by the acronym “CA”) so that in no case there will the possibility of identifying the recruited subject. A psychiatric assessment establishes that patients’ ability to consent is not compromised by their psychopathological status.

## Discussion

This study protocol is composed of two approaches: (1) a cross sectional part that will test whether differences in MLT levels are associated with illness status in BD patients (case–control comparison), and (2) a longitudinal prospective analysis lasting 24 months in which recruited patients will be monitored for the manifestations of mood episodes. Clinical measures and biological material will be collected at baseline in euthymia and when a significant mood disruption will be observed (case-only analysis). In both approaches we will test whether genetic and microbiota variation can modulate the identified associations. Using this approach, we expect to find a significant difference in the plasma levels of MLT and/or some of its precursors between healthy controls and BD patients, and also differences in these biomarkers in BD patients depending on the phase of the disease (depression versus hypomania/mania versus euthymia). Importantly, we also expect to find genetic alterations, in particular in those genes coding for elements of the MLT system, modulating the longitudinal association between the MLT plasma levels and the disease morbidity in BD patients. Very likely, we will identify some typical BD symptomatic clusters that will be more strictly associated with variations in the MLT system. These data could suggest that this group of patients could benefit from pharmacological treatment acting on the MLT system. Furthermore, another important expected result will be the identification of specific microbiota clusters associated with the peripheral variations of MLT, also in relation to the BD disease phase. The combination of extensive phenotypic information with omics data can lead to the discovery of novel, potentially clinically relevant associations, especially when adequate data mining techniques are applied (Breuer et al. 2018).

The study design (cross-sectional in the comparison between controls and BD), however, will not allow us to establish a causality, i.e. if the altered MLT levels precede the development of BD. Therefore, although BD patients will be followed-up longitudinally, this study can be only considered as a proof-of-concept, and thus as a hypothesis generator and identifier of the size of the association between the variables under exam. In spite of these limitations, this study should be able to analyze, in depth and at different levels, the role of the MLT system (MLT and its precursors’ levels and the genetic variability) and its correlation with the microbiota composition in BD pathophysiology. Another limitation may arise by the fact that we will not study the entire 24-h secretion pattern of MLT and the possible variation in its precursors (e.g. by measuring their levels at different time points during the day and the night or measuring the 24-h excretion of 6-sulphatoxymelatonin in the urine) in relationships with the different biological and psychometric markers.

Collectively, these data will help us to better clarify the neurobiology of BD and will be helpful for future projects aimed at the development of new drugs that, by targeting the MLT system, could help improving some of the symptoms of BD. It is important to remember that, to date, there are several pharmacological treatments for BD, but the rate of recurrence of the disease and the toxicity of these drugs are significant. Therefore, there is a great scientific and clinical interest in the development of new drugs, a process for which it is first necessary to better understand the neurobiology of BD.

## Data Availability

The datasets that will be collected during the current study will be available from the corresponding author on reasonable request.
